# 2377: “Pipa” means early death: Obesity and cardiovascular disease (CVD) risks perception, knowledge and behavior among minority NYC cab drivers—A qualitative analysis

**DOI:** 10.1017/cts.2017.266

**Published:** 2018-05-10

**Authors:** Balavenkatesh Kanna, Erida Castro-Rivas, Euripides Roques, Shirley Magabo, Tina Washington, Mohammad Faiz, Namita Tiwari, Andrea Faraci, Edgardo Guzman

## Abstract

OBJECTIVES/SPECIFIC AIMS: More than 2 out of 3 adults in the United States are overweight or obese. Obesity disproportionately affects minority populations. There is limited data on obesity and CVD risks among inner-city minority cab drivers in New York City (NYC). The goal is to study perceptions, knowledge and health behaviors of Hispanic livery cab drivers of NYC that contributes to obesity. METHODS/STUDY POPULATION: We conducted an observational study of focus groups related to perception, knowledge, or behavior among Latino livery cab drivers of NYC. Direct transcription of the taped recordings into concepts were grouped into themes and common themes were categorized. The sample size of the focus groups was based on the saturation point where common themes emerged. RESULTS/ANTICIPATED RESULTS: In total, 25 Latino livery cab drivers were enrolled. Of those, 24 were men. Mean age is 53 years (21–69); body mass index (BMI) is 31 (22.8–38.7) kg/m^2^; 50% had hypertension and 27% had diabetes. Eight dominant themes emerged. Cab drivers were aware of their increased risk for CVD which most of them attributed to work stress, sedentary lifestyle, and poor eating habits “on-the-go”. In particular, they mentioned a tendency of having “Pipa,” a Spanish term denoting increased abdominal girth, which they equated to early death. Family and social support was an important facilitator in changing unhealthy behaviors. DISCUSSION/SIGNIFICANCE OF IMPACT: Our study shows that minority cab drivers are generally obese or overweight and aware of their personal risk factors for CVD including central obesity. Social and family support may be key to improving their lifestyle. An evidenced-based health model that includes family education and decision support will be tested in our next study phase to understand if it can improve body weight.

